# Evaluation of the role of some non-enzymatic antioxidants among Iraqi patients with non-alcoholic fatty liver disease

**DOI:** 10.1515/biol-2022-0881

**Published:** 2024-06-27

**Authors:** Ammar L. Hussein, Dunia T. Nema, Gulboy A. Nasir

**Affiliations:** Department of Biochemistry, College of Medicine, Tikrit University, Tikrit, Iraq; Department of Biomedical Engineering, College of Engineering, Al-Nahrain University, Baghdad, Iraq; College of Agricultural Engineering Sciences, University of Baghdad, Baghdad, Iraq

**Keywords:** nonalcoholic fatty liver disease, antioxidants, coenzyme Q10, vitamin E, vitamin C, oxidative stress

## Abstract

Non-alcoholic fatty liver disease (NAFLD), characterized by hepatic fat accumulation in individuals consuming little or no alcohol, has become highly prevalent globally. Oxidative stress plays a central role in instigating inflammation and cell death pathways driving NAFLD progression. This case–control study aimed to elucidate the association between circulating levels of the pivotal non-enzymatic antioxidants – coenzyme Q10 and vitamins E and C – and liver injury parameters among 60 Iraqi NAFLD patients versus 30 healthy controls. NAFLD diagnosis entailed over 5% hepatic steatosis on ultrasound excluding other etiologies. Patients spanned three age groups: 20–29, 30–39, and 40–49. Substantially diminished antioxidant levels concurrent with elevated alkaline phosphatase enzyme were unveiled in NAFLD patients relative to controls (all *p* < 0.001). Age-based analysis reinforced widespread antioxidant depletion and liver enzyme augmentation across NAFLD patients. Significant correlations also emerged between antioxidants and liver parameters. Our novel observations confirm an antioxidant inadequacy likely perpetuating pathogenic oxidative reactions in NAFLD. Restoring such deficits through lifestyle or therapeutic interventions may confer preventative and disease-modifying value.

## 1 Introduction

Non-alcoholic fatty liver disease (NAFLD) represents the most frequent chronic liver disorder worldwide, with an approximate prevalence of 25% among the global adult population [1,2,3,4,5,6]. The condition is defined by excessive triglyceride accumulation within hepatocytes, prompting hepatic injury, in individuals consuming little or no alcohol [7,8,9,10]. Originally deemed a relatively benign state, simple steatosis can advance to non-alcoholic steatohepatitis (NASH), fibrosis, cirrhosis, and ultimately hepatocellular carcinoma in a subset of patients over years to decades [11,12,13,14]. Nearly 30% of the general population may manifest some degree of NAFLD, rising over 50% among type 2 diabetics in reflection of the intimate pathogenic links with insulin resistance and metabolic dysfunction [1,15,16]. Mirroring the escalating global obesity epidemic, the prevalence of NAFLD is projected to increase markedly over forthcoming years, portending an immense future healthcare burden [1,17,18]. Developing preventative and therapeutic modalities for impeding disease onset and progression is therefore imperative.

A wealth of mechanistic evidence causally implicates oxidative stress in the pathogenesis of NAFLD [19]. Elevated generation of reactive oxygen species overwhelms endogenous antioxidant defenses, instigating inflammatory, fibrogenic, and cell death cascades that drive progressive liver injury [20,21]. Mitochondrial dysfunction, ER stress, immune activation, and other systems constitute sources of augmented reactive oxygen species among multiple preclinical NAFLD models and patients [22]. Accordingly, clinical studies demonstrate depletion of diverse enzymatic and non-enzymatic antioxidants encompassing superoxide dismutase, catalase, glutathione peroxidase, glutathione, vitamin E, vitamin C, and coenzyme Q10 in NAFLD subjects relative to healthy individuals [19,23,24]. Such redox imbalance is believed to arise due to the initial adaptive upregulation of antioxidants attempting to mitigate oxidative damage, followed by eventual overwhelm and decline of these defensive pathways [25]. Hence, strategies to bolster innate antioxidant capacity through lifestyle interventions or therapeutic supplementation represent a biologically rational approach to potentially retard NAFLD progression that warrants further exploration [26,27].

Vitamin E refers to eight structurally related compounds with the α-tocopherol isoform conveying the greatest bioavailability and antioxidant function [28]. Its phenolic hydroxyl moiety readily donates hydrogen to quench lipophilic radicals and reactive species, while other unique mechanisms like protein kinase C modulation elicit anti-inflammatory actions [29,30,31]. Animal studies of experimental NASH demonstrate vitamin E restoration of glutathione alongside reductions in oxidative damage, inflammation, stellate cell activation, and histological fibrosis versus disease controls [32]. The hydrophilic vitamin C directly scavenges diverse reactive oxygen species and also sparingly regenerates lipid-soluble antioxidants like vitamin E to disrupt deleterious oxidation reactions [33]. Epidemiological studies reveal an inverse relationship of vitamin C intake with human NAFLD severity, although occasional inconsistent outcomes have been reported [34,35,36]. Coenzyme Q10 is a crucial electron carrier that facilitates mitochondrial ATP generation and participates in membrane stabilization and beta-oxidation [37]. Through such properties, coenzyme Q10 elevation putatively suppresses lipotoxic liver injury, oxidative stress, and inflammation in NAFLD, with mixed clinical findings thus far [38,39,40].

In efforts to provide further human evidence regarding perturbations in redox homeostasis arising during NAFLD, we examined circulating levels of the above described, biologically active non-enzymatic antioxidants – coenzyme Q10 and vitamins E and C – alongside the hepatic injury marker alkaline phosphatase among 60 Iraqi NAFLD patients compared to 30 healthy controls. Determining whether antioxidant deficits associated with this high-risk condition could substantiate a basis for restoring such reserves nutritionally or pharmaceutically to mitigate pathogenic liver oxidative damage.

## 2 Materials and methods

### 2.1 Study design and participants

This case–control study enrolled 60 NAFLD patients alongside 30 healthy volunteers at Tikrit Teaching Hospital (Iraq) over a 3-month interval from October to December 2023. NAFLD diagnosis entailed ultrasonographic evidence of hepatic steatosis exceeding 5% of hepatocytes excluding secondary causes such as significant alcohol consumption, viral infection, or drugs per established criteria [39]. Patients spanned three age brackets: 20–29 years (*n* = 9), 30–39 years (*n* = 17), and 40–49 years (*n* = 34). Controls consisted of age-matched healthy adults without liver disease.


**Informed consent:** Informed consent has been obtained from all individuals included in this study.
**Ethical approval:** The research related to human use has been complied with all the relevant national regulations, institutional policies and in accordance with the tenets of the Helsinki Declaration, and has been approved by the Ethical Committee of College of Medicine, Tikrit University (203 on 06.02.2023).

### 2.2 Sample analysis

Overnight fasting blood samples were collected from participants and centrifuged to isolate serum. ELISA techniques quantified concentrations of coenzyme Q10, vitamin E, vitamin C (indices of antioxidant status), and alkaline phosphatase (marker of hepatic injury) per kit protocols.

### 2.3 Statistical analysis

Data were expressed as mean ± standard deviation. Significant between-group differences were evaluated by independent samples’ *t*-test or Mann–Whitney *U* test as appropriate, with *p* < 0.05 deemed statistically significant. Pearson’s correlation test determined linear associations between parameters. Statistical Package for Social Sciences software version 28.0 facilitated analyses.

## 3 Results

### 3.1 Demographic characteristics of study participants

Table 1 displays a comparative summary of demographic and clinical features between NAFLD patients and healthy controls. Most patients were middle-aged and obese in contrast to the young, normal-weight predominance among control adults.

**Table 1:** Demographic and anthropometric characteristics of NAFLD patients versus healthy controls

Variables	Groups	Patient	Control	Chi-square	*p* value
Age groups	20–29 years	9	12	8.4	0.015
	30–39 years	17	9		
	40–49 years	34	9		
BMI	Normal weight	2	18	47.8	0.00001
	Overweight	13	10		
	Obesity	45	2		
Sex	Male	30	15	0	1
	Female	30	15		

### 3.2 Circulating antioxidants and liver enzyme among cases versus controls

As shown in Table 2, relative to healthy controls, Iraqi NAFLD patients displayed markedly reduced serum levels of coenzyme Q10 (29.5 ± 5.2 vs 18.3 ± 5.3 μmol/L), vitamin E (58.9 ± 18.2 vs 43.1 ± 22.0 μmol/L), and vitamin C (13.2 ± 2.7 vs 6.5 ± 2.7 mg/L) (all *p* < 0.001), indicating a gross systemic antioxidant deficit. Conversely, the hepatic injury indicator alkaline phosphatase was notably elevated among NAFLD patients compared to controls (101.7 ± 15.7 vs 74.4 ± 5.1 U/L, *p* < 0.001).

**Table 2:** Comparison of circulating antioxidants and alkaline phosphatase levels between NAFLD patients and healthy controls

Variable	Patients, mean ± SD	Control, mean ± SD	*p*-value
CoQ10	18.32 ± 5.32	29.50 ± 5.18	*p* < 0.001
Vitamin E	43.14 ± 21.96	58.90 ± 18.20	
Vitamin C	6.54 ± 2.71	13.20 ± 2.66	
ALP	101.68 ± 15.72	74.30 ± 5.10	

### 3.3 Stratified analysis of antioxidants and liver enzymes by age

Further age-wise scrutiny of cases versus controls, as delineated in Tables 3–5, reinforced widespread reductions in examined antioxidants among Iraqi NAFLD patients across all age groups, ranging from young adults to middle-aged individuals. Concurrently, alkaline phosphatase also remained markedly elevated in patients compared to controls across the age spectrum. The sole outlier was vitamin E in the youngest (20–29 years) patient subgroup, which did not significantly differ versus corresponding controls. Nevertheless, collectively these observations verify gross antioxidant deficits and liver enzyme augmentation manifesting quite ubiquitously from early in the natural history of NAFLD regardless of age.

**Table 3:** Comparison of circulating antioxidants and alkaline phosphatase among 20–29-year-old NAFLD patients versus controls

Variables	Patients, mean ± SD, *N* = 8	Control, mean ± SD, *N* = 10	*p*-value
**(20 – 29) years – G1**			
CoQ10	19.59 ± 6.48	28.96 ± 4.50	0.002
Vitamin E	61.28 ± 31.37	56.89 ± 15.92	0.724
Vitamin C	6.73 ± 1.74	13.13 ± 1.82	0.001
ALP	97.43 ± 12.82	75.25 ± 5.52	0.001

**Table 4:** Comparison of circulating antioxidants and alkaline phosphatase among 30–39-year-old NAFLD patients versus controls

Variables	Patients, mean ± SD, *N* = 8	Control, mean ± SD, *N* = 10	*p*-value
**(30–39) years – G2**			
CoQ10	19.089 ± 6.329	30.905 ± 5.248	0.001
Vitamin E	33.361 ± 8.672	62.730 ± 22.559	0.002
Vitamin C	6.73 ± 1.74	13.13 ± 1.82	0.001
ALP	97.43 ± 12.82	75.25 ± 5.52	0.001

**Table 5:** Comparison of circulating antioxidants and alkaline phosphatase among 40–49-year-old NAFLD patients versus controls

Variables	Patients, mean ± SD, *N* = 15	Control, mean ± SD, *N* = 10	*p*-value
**(40–49) years – G3**			
CoQ10	17.74 ± 4.65	28.61 ± 6.09	0.001
Vitamin E	43.19 ± 21.38	57.10 ± 16.85	
Vitamin C	6.61 ± 3.24	13.53 ± 2.99	
ALP	101.20 ± 15.37	75.25 ± 5.14	

### 3.4 Interrelationships between oxidative stress markers and liver function

**Figure 1:**
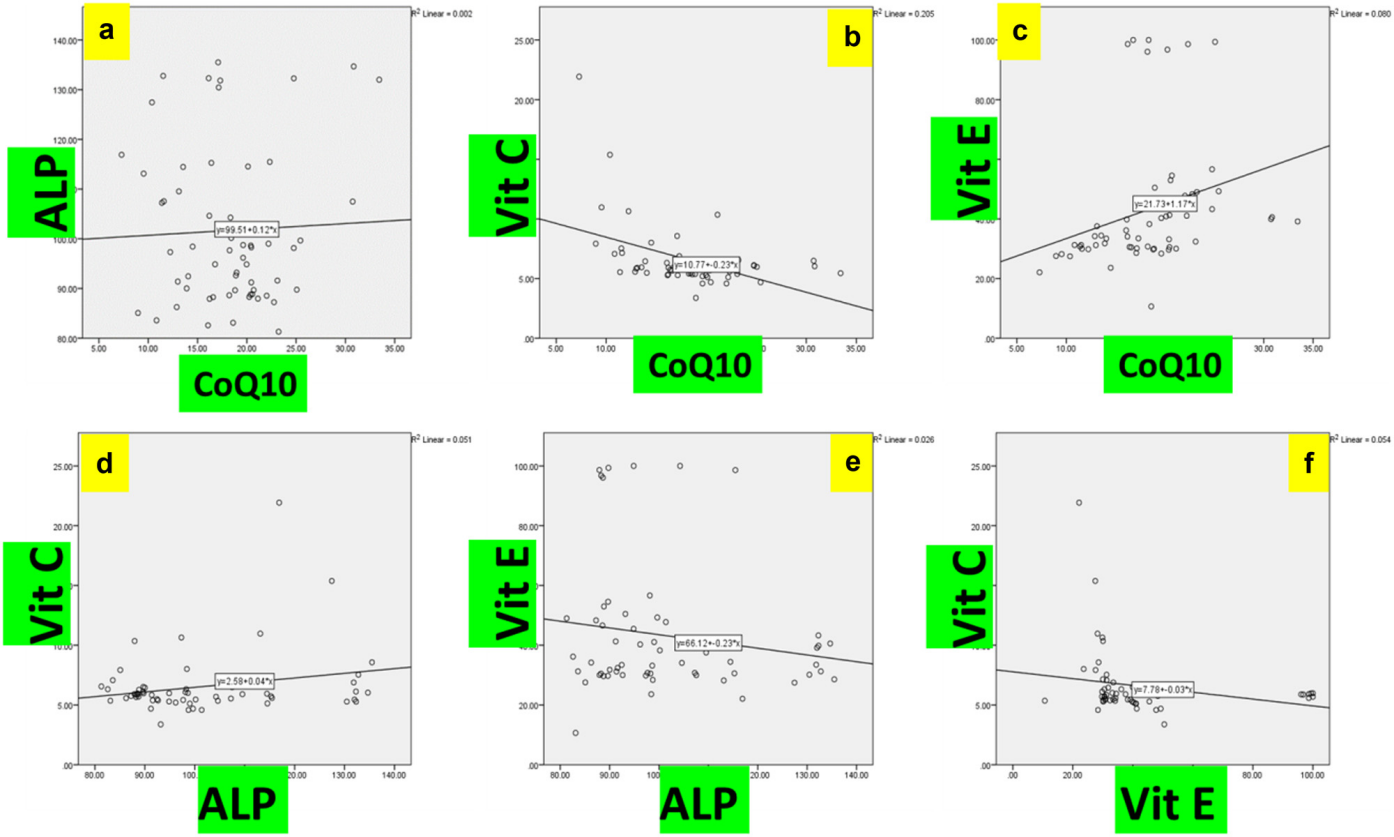
Correlative analysis between circulating antioxidants and liver enzyme in Iraqi NAFLD patients. (a) Association between coenzyme Q10 and alkaline phosphatase levels. (b) Association between coenzyme Q10 and vitamin C levels. (c) Association between coenzyme Q10 and vitamin E levels. (d) Association between alkaline phosphatase and vitamin C levels. (e) Association between alkaline phosphatase and vitamin E levels. (f) Association between vitamin C and vitamin E levels.

Correlation analysis, as shown in Figure 1, unveiled no significant linear association between coenzyme Q10 and alkaline phosphatase amongst Iraqi NAFLD patients (*p* = 0.45). However, circulating coenzyme Q10 manifested a robust inverse correlation with vitamin C levels (*r* = −0.7, *p* = 0.001). Significant positive correlations were apparent between coenzyme Q10 and vitamin E (*r* = 0.7, *p* = 0.001), alkaline phosphatase and vitamin C (*r* = 0.4, *p* = 0.03), alkaline phosphatase and vitamin E (*r* = 0.4, *p* = 0.03), along with vitamins C and E (*r* = −0.5, *p* = 0.04).

## 4 Discussion

In this case–control study, we report markedly depleted circulating concentrations of the pivotal non-enzymatic antioxidants; coenzyme Q10, vitamin E, and vitamin C paralleling elevated alkaline phosphatase liver enzyme among Iraqi NAFLD patients relative to healthy controls. Age-stratified analysis reinforced pervasive antioxidant deficits and liver function abnormalities manifesting quite ubiquitously regardless of age variation. Significant inter-relationships were also unveiled between these redox homeostatic markers and hepatic parameter. Collectively, our novel observations verify perturbation of antioxidant capacity likely perpetuating pathogenic oxidative damage that drives NAFLD progression. Strategies to rectify such inadequacies nutritionally or pharmacologically may offer preventative and therapeutic value.

Excess intrahepatic lipid accumulation incites complex pathogenic cascades encompassing lipotoxicity, aberrant metabolites, mitochondrial dysfunction, ER stress, and immune activation that boost the production of reactive oxygen species and nitrogen species [22,41]. Resultant oxidative stress overwhelms endogenous antioxidant defenses to trigger inflammatory, apoptotic, and fibrogenic pathways critical to NASH and fibrosis development [20]. Accordingly, both experimental models and human studies demonstrate the depletion of diverse enzymatic and non-enzymatic antioxidants in NAFLD subjects [23,26,42,43]. Our observations confirm significant attrition of circulating coenzyme Q10, vitamin E, and vitamin C levels amongst Iraqi NAFLD patients, likely perpetuating such hepatic oxidative injury. Vitamin E demonstrates potent radical-scavenging activity and also enhances glutathione recycling, suppresses inflammatory signaling, and reduces oxidative damage, immune infiltration, and fibrosis progression in experimental NASH [32,44,45]. Vitamin C directly deactivates diverse reactive oxygen species and sparingly regenerates membrane antioxidant vitamin E following oxidative stress [33]. Coenzyme Q10 facilitates mitochondrial respiration and correspondingly lowers ROS generation, while also stabilizing membranes [37]. Augmenting the availability of such innate compounds through dietary or supplemental means may therefore confer preventative and therapeutic advantages by mitigating oxidative reactions underlying NAFLD pathogenesis – a premise warranting further scrutiny. In addition to the impact of coenzyme Q10 on liver, it carries potential beneficial effects against drug toxicity [46,47] or improvement of other chronic conditions [48,49,50].

Interestingly, whereas coenzyme Q10 did not associate with alkaline phosphatase levels, significant correlations existed between the vitamins and enzyme. Differential subcellular partitioning probably determines such divergent interrelationships. Nevertheless, collectively restoring depressed antioxidant reserves may impart broader therapeutic benefit. Our age-defined analysis notably reinforced an overt antioxidant deficit manifesting quite pervasively from younger adulthood onwards regardless of demographic variation. This suggests that redox imbalance arises relatively early in NAFLD and represents an integral component of pathogenic progression rather than solely a late secondary phenomenon. Addressing such inadequacies proactively could therefore offer advantage.

This study has certain limitations meriting consideration. Our cohort size was modest, preventing more nuanced patient stratification by gender, body mass index (BMI), or other factors. Dietary habits, physical activity profiles, smoking status, and comorbid conditions were not captured but may conceivably impact outcomes. The histological examination represents the gold standard for accurately staging NAFLD severity. Nevertheless, our findings provide clinical evidence substantiating the pronounced depletion of key non-enzymatic antioxidants and verifying oxidative stress contributions toward early NAFLD pathogenesis. Further studies in larger populations would valuably validate and extend these observations.

## 5 Conclusion

In conclusion, relative to healthy controls, Iraqi NAFLD patients exhibited significantly reduced circulating concentrations of the pivotal antioxidants coenzyme Q10, vitamin E, and vitamin C complementing elevated alkaline phosphatase liver enzyme. Age-stratified analysis reinforced pervasive antioxidant inadequacy and liver dysfunction progressing from young adulthood regardless of demographic variability. Significant inter-relationships were also unveiled between these redox-homeostatic markers and enzyme parameter. Collectively, our novel observations confirm perturbation of antioxidant capacity likely perpetuating pathogenic oxidative reactions underlying NAFLD onset and progression. Strategies to rectify such deficits through lifestyle and therapeutic interventions may offer preventative and disease-modifying value that warrants further evaluation.

Several limitations should be considered when interpreting findings from this study. The study is a case–control, which can only establish associations and not causality. Longitudinal studies or randomized controlled trials would provide stronger evidence for the role of antioxidants in NAFLD. The cohort size was modest, restricting extensive demographic-based stratifications. Detailed dietary information, smoking patterns, physical activity profiles, and comorbidity data were not captured. The study focused on the levels of coenzyme Q10 and vitamins E and C as non-enzymatic antioxidants. There are other antioxidants that could also play a role in NAFLD, and their inclusion in future studies would provide a more comprehensive understanding of antioxidant status in this condition. Moreover, only three age groups included in the study and other age groups need to be investigated. Histopathology represents the diagnostic gold standard for precisely staging NAFLD severity and inflammation, rather than relying exclusively on imaging criteria. Nonetheless, such limitations apply equally to cases and controls. Hence, this study provides novel clinical evidence substantiating oxidative stress mechanisms in early human NAFLD pathogenesis.
